# Inhibitory effect of *Actinidia arguta* on mutagenesis, inflammation and two-stage mouse skin tumorigenesis

**DOI:** 10.1186/s41021-016-0053-9

**Published:** 2016-11-01

**Authors:** Mari Nishimura, Yuma Okimasu, Naoko Miyake, Misako Tada, Ryoko Hida, Tomoe Negishi, Sakae Arimoto-Kobayashi

**Affiliations:** 1 Graduate School of Medicine, Dentistry and Pharmaceutical Sciences, Tsushima, Okayama 700-8530 Japan; 2 Faculty of Pharmaceutical Sciences, Okayama University, Tsushima, Okayama 700-8530 Japan

**Keywords:** *Actinidia arguta*, Chemoprevention, Antimutagenesis, DNA adduct, Radical scavenging, Skin cancer, Enzyme inhibition

## Abstract

**Background:**

*Actinidia arguta*, known as sarunashi in Japan, is a vine tree native to east-Asia, including Japan, that produces small fruit rich in anthocyanins, catechins, vitamin C, chlorophyll, beta-carotene and other polyphenols.

**Results:**

Our study revealed the inhibitory effect of the juice of *A. arguta* (arguta-juice) toward the mutagenicity of food-derived carcinogens and polycyclic aromatic hydrocarbons using the Ames test, and antioxidant activity of arguta-juice as determined using a free radical scavenging assay. The formation of DNA adducts in liver of mice fed 2-amino-3,8-dimethylimidazo[4,5-*f*]quinoxaline (MeIQx) decreased significantly following administration of arguta-juice. The preventive effect of arguta-juice on the induction of inflammation of mouse ear by 12-*O*-tetradecanoylphorbol-13-acetate (TPA) was revealed. The anti-carcinogenic effect of a topically applied partially purified fraction of *A. arguta* was revealed on skin tumorigenesis in mice induced by treatment with 7,12-dimethylbenz(a)anthracene and TPA. In an effort to reveal the mechanisms for antimutagenicity of arguta-juice, effects on the enzymes that metabolize xenobiotics were examined. Combined effects comprising i) inhibition of the metabolic activation of mutagens with phase I enzymes, but ii) no prevention on the activity of phase II detoxification enzyme, UGT, were observed. We also investigated the characterization and partial purification of the antimutagenic components in *A. arguta*, which suggested that the components in *A. arguta* responsible for the antimutagenicity were water-soluble, heat-labile phenolic compounds.

**Conclusions:**

These results suggested that components in *A. arguta* are attractive candidates for potential use as chemopreventive agents.

## Background

Tumor progression is driven by a sequence of randomly occurring mutations that affect the genes controlling cell proliferation [1]. A significant portion of human cancers can be attributed to the consumption of foodstuffs and smoke-contaminated mutagenic carcinogens. Exposure to environmental mutagens including food-borne heterocyclic amines, polyaromatic hydrocarbons and fungal toxins act through their ability to mutate DNA and/or promote tumorigenesis [2]. Endogenous oxidative stress, including the production of reactive oxygen radicals in mitochondria through aerobic metabolic processes, can also result in oxidative damage and mutations in DNA, which has been implicated in chronic diseases [3]. Inflammation also contributes to carcinogenesis [4]. Under inflammatory conditions, reactive oxygen species are generated from inflammatory cells that can result in oxidative DNA damage. The DNA damage can cause mutations and has been implicated in the initiation and/or promotion of inflammation-mediated carcinogenesis.

Dietary factors play a major role in a variety of human disorders and are capable of affording protection against diseases including cancer [2]. Diets rich in fruit and vegetables are known to decrease the risk of many cancers. Fruit and vegetables rich in polyphenolics have been studied due to their effect on reducing oxidative stress and several chronic diseases [5, 6]. In our recent study, the juice of *Vitis coignetiae*, which is rich in polyphenols, significantly inhibited the mutagenicity and clastogenicity of several mutagens using the Ames test and micronucleus test [7]. Topical application of the juice of *V. coignetiae* significantly decreased the incidence and multiplicity of tumors in mice skin using a two-stage tumorigenesis protocol [8]. *Actinidia arguta*, known as sarunashi in Japan and hardy kiwi in English name, is a vine tree native to east-Asia, including Japan [9], that produces small fruit consumed in the form of fresh fruit and jam. The fruit is rich in anthocyanins [10], catechins [11], vitamin C [12], chlorophyll, beta-carotene [13] and other polyphenols [14, 15]. Antioxidant activity of *A. arguta* has been reported in recent studies [16–18], and components in *A. arguta* have been reported to possess anti-allergic activity [19–21].

In the present study, we investigated the effect of the juice of the fruit of *A. arguta* on the antimutagenic activity of food-derived heterocyclic amines and other carcinogens using the Ames test, antioxidant activity using a free radical scavenging assay, and DNA adduct formation induced by 2-amino-3,8-dimethyl-3*H*-imidazo[4,5-*f*]quinoxaline (MeIQx). The effect of the juice of *A. arguta* on the induction of inflammation of mouse ear by 12-*O*-tetradecanoylphorbol-13-acetate (TPA) was examined, and characterization and partial purification of antimutagenic components present in *A. arguta* juice were investigated. Furthermore, we evaluated the antitumorigenic activity of a partially purified fraction of the juice of *A. arguta* using a two-stage mouse skin tumorigenesis protocol.

## Methods

### Materials and animals

Fruits of *Actinidia arguta* (Hardy kiwifruits) and *Actinidia deliciosa* cv. Hayward (kiwifruits) were used in this experiment (not use any other parts, leaves, flowers, roots etc.). Fruit of *A. arguta* was harvested in the village Shinjo (Okayama, Japan) and stored at −20 °C until use. Prior to the experiments, the fruit was defrosted and then squeezed with a press-squeeze to obtain the juice (hereafter referred to as arguta-juice). Squeezed juice was stored at −20 °C until use. Crops comprised a blend of *A. arguta* 'Mitsu-ko' and *A. arguta* 'Hou-ko', unless otherwise indicated. For the comparison between cultivars, fruit was separately harvested from vines of *A. arguta* 'Mitsu-ko', *A. arguta* 'Hou-ko', and a wild vine of *A. arguta* found on a hillside in the village Shinjo, the latter hereafter referred to as *A. arguta* 'Wild-S'. Half of the crops from *A. arguta* 'Mitsu-ko', 'Hou-ko' and ' Wild-S ' were separately squeezed and stored at −20 °C within 24 h after harvesting (hereafter referred to as fresh samples). The other half of the crops were stored at room temperature for three days, squeezed, and then stored at −20 °C (hereafter referred to as 3rd-day samples). For the Ames test, arguta-juice was centrifuged at 2200 g for 15 min, and the supernatant was sterilized by filtration. The filtrate was used for the Ames test without further processing or purification. For the in vivo experiments involving DNA adduct formation, arguta-juice was freeze-dried and then dissolved in distilled water to one-third or equal volume of the original juice volume, hereafter referred to as "arguta-juice (x3)" and "arguta-juice (x1)", respectively. For the experiments involving topical application to mice, arguta-juice was freeze-dried and then dissolved in 66 % acetone to one-third or equal volume of the original juice volume, hereafter referred to as "arguta-solution (x3)" and "arguta-solution (x1)", respectively. The total amount of phenolics in arguta juice was measured using the method described by Singleton and Rossi [22]. The quantitative calibration was performed with standard solutions of gallic acid. Kiwifruit (*Actinidia deliciosa* cv. Hayward) harvested in Okayama was purchased in local stores in Okayama in 2008, and juice (hereafter referred to as "deliciosa-juice") was obtained as described above.

Aflatoxin B1 (CAS 1162-65-8), 2-amino-3,8-dimethyl-3*H*-imidazo[4,5-*f*]quinoxaline (MeIQx, CAS 77500-04-0), 2-amino-1-methyl-6-phenylimidazo[4,5-*b*]pyridine (PhIP, CAS 105650-23-5) and 3-amino-1-methyl-5*H*-pyrido[4,3-*b*]indole (Trp-P-2, CAS 72254-58-1), benzo(a)pyrene (CAS 50-32-8), 7,12-dimethylbenz(a)anthracene (DMBA, CAS57-97-6), and 12-*O*-tetradecanoylphorbol-13-acetate (TPA, CAS16561-29-8) were purchased from Wako Pure Chemical Co. Ltd. (Osaka, Japan). Polyvinylpolypyrrolidone (PVPP) was purchased from Nacalai Tesque, Inc. Other chemicals used were commercial products of reagent grade. *Salmonella enterica subspecies I, serovar Typhimurium* (*Salmonella typhimurium*) strain TA98 [*hisD3052 ΔuvrB gal bio chl1005 rfa1001/pKM101*], and strain TA100 [*hisG46 ΔuvrB gal bio chl1005 rfa1001/pKM101*] were gifts from Dr. B.N. Ames of the University of California, Berkeley CA [23]. The supernatant fraction of rat liver homogenate (S9) was prepared from male Sprague–Dawley rats that had been induced by the administration of polychlorinated biphenyl (PCB54, with a chlorine content of 54 %, Tokyo Kasei, Tokyo). The protein content of the S9 fraction was 43.0 mg/mL. Sprague–Dawley rats and ICR mice were obtained from Japan SLC, Inc (Hamamatsu, Japan), C57BL/6 N mice were obtained from Charles River Japan (Atsugi, Japan), and female SENCAR mice were born in our laboratory. All experiments were performed in accordance with the Safety Guidelines of Okayama University and the Japanese Government Management Law for toxic chemicals (No. 303).

### Antimutagenicity test

The inhibitory effect of arguta-juice on mutagenicity induced by MeIQx (60 pmol), Trp-P-2 (100 pmol), PhIP (1.0 nmol), aflatoxin B1 (3.0 nmol), benzo(a)pyrene (10 nmol) and DMBA (50 nmol) were investigated using the Ames test [23]. An original sample of arguta-juice, not dissolved after freeze-drying, was used for the assay. MeIQx, PhIP, Trp-P-2 and aflatoxin B1 were assayed using *S. typhimurium* TA98, and benzo(a)pyrene and DMBA were assayed using *S. typhimurium* TA100 in the presence of S9. The effect of the juice on mutagenicity was examined as previously described [7]. Experiments were performed in triplicate. Mutagenic activity (%) as shown in the figures was derived as follows:

$$ 100\times \left[\left(\mathrm{revertants}\ \mathrm{in}\ \mathrm{the}\ \mathrm{presence}\ \mathrm{of}\ \mathrm{juice}\right)\ \hbox{--}\ \left(\mathrm{spontaneous}\ \mathrm{revertants}\right)\right]/\left[\left(\mathrm{revertants}\ \mathrm{in}\ \mathrm{the}\ \mathrm{absence}\ \mathrm{of}\ \mathrm{juice}\right)\ \hbox{--}\ \left(\mathrm{spontaneous}\ \mathrm{revertants}\right)\right]. $$

### Detection of DNA adducts in mice

C57BL/6 N mice (males, 6 weeks old) were randomly divided into four groups each comprising 8 mice. For investigation of the influence of arguta-juice on MeIQx-DNA adduct formation, 1.5 mL of arguta-juice (x1 or x3) and/or MeIQx (final concentration 0.005 %/diet/day) was mixed with the control diet (MF powder, Oriental Yeast Co., Ltd., Tokyo, Japan; 1.5 g) to yield a diet-paste (3 g). For two days, C57BL/6 N mice received diet-paste mixed with either water (Group 1), arguta-juice (x1) (Group 2), or arguta-juice (x3) (Groups 3 & 4) without MeIQx. Then, for three days, mice were given a diet (3 g/mouse) mixed with MeIQx (Group 1), arguta-juice (x1) and MeIQx (Group 2), arguta-juice (x3) and MeIQx (Group 3), or arguta-juice (x3) (Group 4). On day 6, mice were sacrificed by cervical dislocation and extracted tissues were washed with ice-cold KCl (0.15 M) and then frozen in liquid nitrogen. Samples were stored at −80 °C until use. The amount of heterocyclic amine-DNA adduct in the DNA of treated mice tissues shown in Table 1 was determined by modified adduct-intensification analysis using the ^32^P-postlabeling method [24].

### Effect on enzyme activity

Arguta-juice was dissolved in water after freeze-drying and used for the assay. Sample volumes refer to mL equivalents of the original juice. The activity of cytochrome P450 1A1 (CYP 1A1) and cytochrome P450 1A2 (CYP1A2) was measured as ethoxyresorufin*-O*-deethylase (EROD) and methoxyresorufin-*O*-demethylase (MROD) activity, respectively, according to the methods reported by Iwata et al. [25]. Fluorescence intensity was measured at λ ex 535 nm and λ em 582 nm. Enzyme activity was expressed as relative fluorescent intensity, which was calculated for each reaction by normalizing the control to 100 %. The fluorescence of metabolites was not altered by the presence of pigments in the juice samples at the examined concentrations.

The activity of uridine 5'-diphosphoglucuronosyltransferase (UDP-glucuronosyltransferase, UGT) was determined by measuring the reduction in absorbance of 4-nitrophenol at 405 nm as described by Jakoby [26]. Enzyme activity was expressed as relative fluorescent intensity, which was calculated for each reaction by normalizing the control to 100 %. Glutathione *S*-transferase (GST) activity was determined by the method of Habig et al. [27], using 1-chloro-2,4-dinitrobenzene as a substrate. The influence of arguta-juice on GST activity was investigated by examining the formation of Glutathione conjugate, and measured by monitoring the optical density at 340 nm. Enzyme activity as shown was expressed as a relative intensity, which was calculated for each reaction by normalizing the control to 100 %. Experiments were performed in triplicate.

### Effect on TPA-induced acute edema on the surface of mouse ears with topical application of arguta-solution

Six-week-old male mice (ICR) weighing 16–18 g were divided into five groups for the experiment. Mice in Group 1 (TPA-treated control) and Group 4 (acetone-treated control) received 20 μL of 66 % acetone applied on the surface of both ears. Mice in Group 2 received 20 μL of arguta-solution (x1) dissolved in 66 % acetone, and mice in Groups 3 and 5 received 20 μL of arguta-solution (x3) dissolved in 66 % acetone, applied on the inner and outer surface of both ears, respectively. After 30 min, 1.7 nmol of TPA dissolved in acetone was applied on the surface of both ears of mice in Groups 1–3 for the induction of topical acute edema. Mice in Groups 4 and 5 received 100 % acetone without TPA. Thirty minutes following TPA treatment mice were sacrificed and both ears were removed. Circular sections (6 mm in diameter) of all treated ears were punched out using a cork borer and weighed. Edema was quantified as the weight difference between treatments. Anti-inflammatory activity was evaluated as the percentage of edema reduction/induction in the treated mouse relative to the control mouse. Inhibition (%) as shown in Table 2 was derived as follows:

$$ 100\times \Big[\left(\mathrm{average}\ \mathrm{weight}\ \mathrm{of}\ \mathrm{ear}\ \mathrm{punch}\ \mathrm{treated}\ \mathrm{with}\ \mathrm{T}\mathrm{P}\mathrm{A}\ \mathrm{and}\ \mathrm{arguta}\hbox{-} \mathrm{solution}\right)\hbox{-} \left(\mathrm{average}\ \mathrm{weight}\ \mathrm{of}\ \mathrm{ear}\ \mathrm{punch}\ \mathrm{treated}\ \mathrm{with}\mathrm{out}\ \mathrm{T}\mathrm{P}\mathrm{A}\ \mathrm{or}\ \mathrm{arguta}\hbox{-} \mathrm{solution}\right)\left]/\right[\left[\left(\mathrm{average}\ \mathrm{weight}\ \mathrm{of}\ \mathrm{ear}\ \mathrm{punch}\ \mathrm{treated}\ \mathrm{with}\ \mathrm{T}\mathrm{P}\mathrm{A}\ \mathrm{with}\mathrm{out}\ \mathrm{arguta}\hbox{-} \mathrm{solution}\right)\ \hbox{-}\ \left(\mathrm{average}\ \mathrm{weight}\ \mathrm{of}\ \mathrm{ear}\ \mathrm{punch}\ \mathrm{treated}\ \mathrm{with}\mathrm{out}\ \mathrm{T}\mathrm{P}\mathrm{A}\ \mathrm{or}\ \mathrm{arguta}\hbox{-} \mathrm{solution}\right)\right]. $$

### Fractionation of antimutagenic and radical scavenging components in arguta-juice

**Fig. 1:**
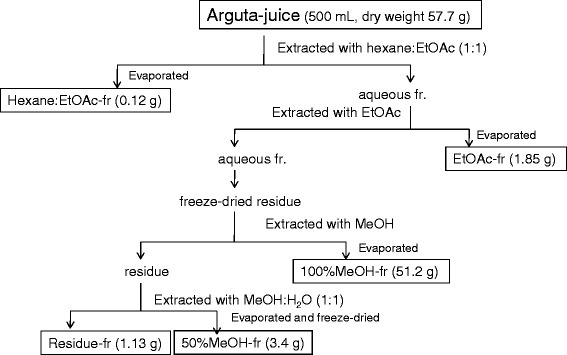
Flow chart detailing the isolation of an active component from arguta-juice. Arguta-juice was subjected to a sequence of solvent-solvent partitioning. The aqueous fraction after EtOAc extraction was freeze-dried and the residue was extracted with methanol. Weight in parentheses of the dry weight of each fraction from 500 mL of original juice

Fractionation of antimutagenic and radical scavenging components in arguta-juice is outlined in Fig. 1. Briefly, arguta-juice was subjected to a sequence of solvent-solvent partitioning, i.e., hexane: ethyl acetate (EtOAc) (1:1) extraction and EtOAc extraction (hereafter referred to as Hexane: EtOAc-fr and EtOAc-fr, respectively). The aqueous fraction after EtOAc extraction was freeze-dried and the residue was extracted with methanol (MeOH) and then methanol: water (1:1) (hereafter referred to as 100%MeOH-fr and 50%MeOH-fr, respectively). Hexane:EtOAc-fr, EtOAc-fr and 100%MeOH-fr were each evaporated under reduced pressure to dryness. 50%MeOH-fr was evaporated to remove methanol and then freeze-dried. The obtained residues were weighed. For the in vitro assay using the Ames test and 2,2-diphenyl-1-picrylhydrazyl (DPPH) assay, residues were dissolved in sterilized water. For the in vivo experiments involving topical application to mice, freeze-dried 50%MeOH-fr was dissolved in 50 % acetone to one-fifth or one-tenth volume of the original juice volume, hereafter referred to as "50%MeOH-fr -solution (x5)" and "50%MeOH-fr -solution (x10)", respectively. The antimutagenicity of each fraction was monitored for its effect on MeIQx (60 pmol/plate) mutagenicity assayed in the presence of S9 with *S. typhimurium* TA98. The free radical scavenging activity of arguta-juice and each fraction was measured using the DPPH scavenging assay [28]. The inhibitory percentage of DPPH was calculated according to the following equation: Scavenging effect (%) = (optical density (OD) of control – OD of sample/OD of control) × 100. The concentration of vitamin C was measured by the indophenol method [29]. The experiments were performed in triplicate.

### Effect on tumor promotion following topical application of 50%MeOH-fr

Six-week-old female SENCAR mice were randomly divided into four groups each comprising 12 or 13 mice. The dorsal side of the skin of mice was shaved using an electric hair cutter at least two days prior to treatment. Skin tumors were induced chemically using a standard 2-stage initiation-promotion protocol [30]. The initiation was accomplished using a single topical application of DMBA (2.5 mg/animal, dissolved in 0.1 mL acetone) on the dorsal shaved skin of mice in Groups 1–4. Seven days later, mice in Group 1 (DMBA treated control) were treated topically with 0.1 mL of 66 % acetone, while mice in other groups were treated with either 50%MeOH-fr-solution (x5) (Group 2) and/or 50%MeOH-fr-solution (x10) (Groups 3 and 4) dissolved in 0.1 mL of 50 % acetone per application. Thirty minutes following treatment, mice in Groups 1–3 were treated topically with TPA (1.7 nmol) dissolved in 0.1 mL acetone, while mice in Group 4 were treated topically with 0.1 mL acetone as a substitute for the TPA solution. The TPA and/or 2-dose 50%MeOH-fr treatments were repeated twice per week until the termination of the experiment 20 weeks from the start of the DMBA application. During the period, the tumor yield and incidence were recorded weekly, and tumors >1 mm in diameter were included in the cumulative total if they persisted for at least 2 weeks. The data are expressed as the percentage of mice with tumors and number of tumors per mouse, and are plotted as a function of weeks during the test.

### Effect of heat and PVPP treatment on the antimutagenicity of arguta-juice and 50%MeOH-fr

For the experiments of heat treatment, sample was placed in a water bath at 90 °C for 10 min and then cooled on ice. Heated juice was then centrifuged at 2200 g for 15 min and the supernatant was sterilized by filtration. For the experiments involving PVPP treatment, 1-mL sample was mixed with 2 mg of PVPP and then shaken for 30 min. The mixture was then centrifuged and the supernatant was sterilized by filtration. The total amount of phenolics was determined before and after the PVPP treatment. The antimutagenicity of the treated and untreated samples was monitored for the effect on MeIQx (60 pmol/plate) mutagenicity.

### Statistical analyses

Data are expressed as means ± standard deviation for each data point as indicated in each Figure or Table. Statistical analyses were performed using Dunnett's test and Kaplan-Meier method with KaleidaGraph (Synergy Software, Reading, PA), Excel add-in soft (SSRI CO. Ltd, Tokyo, Japan), and JMP (JMP Japan, Tokyo, Japan).

## Results

### Antimutagenicity of arguta-juice

**Fig. 2:**
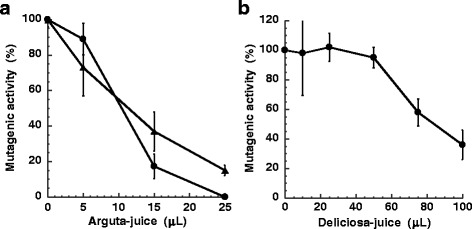
Effect of arguta-juice (**a**) and deliciosa-juice (**b**) on the mutagenicity of MeIQx (60 pmole). An original sample of arguta-juice, not dissolved after freeze-drying, was used for the assay. MeIQx was assayed using *S. typhimurium* TA98 in the presence of S9. The number of His^+^ revertants per plate found in the absence of juice was 1010 ± 63 for 60 pmol of MeIQx. Harvesting of the fruit of *A. arguta* were performed in 2008 (circles) and 2010 (triangles). Harvesting of the fruit of *A. deliciosa* was performed in 2008

**Fig. 3:**
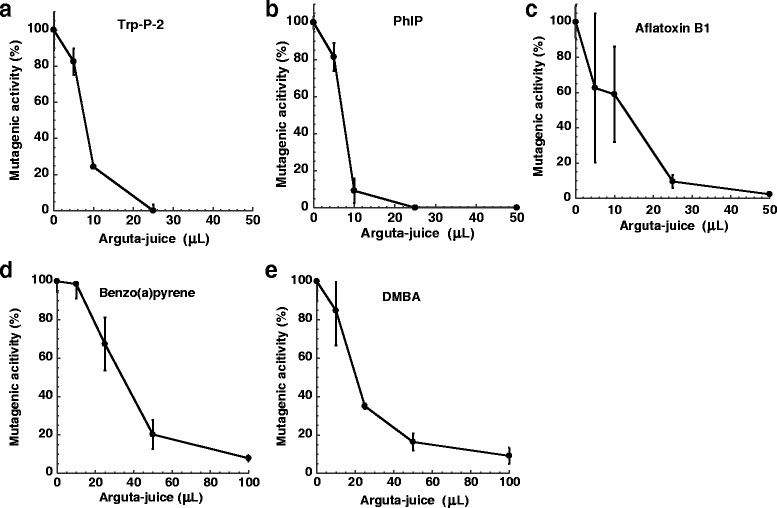
Effect of arguta-juice on the mutagenicity of Trp-P-2 (**a**), PhIP (**b**), aflatoxin B1 (**c**), benzo(a)pyrene (**d**), and DMBA (**e**). Harvesting of the fruit of *A. arguta* was performed in 2008. MeIQx, PhIP, Trp-P-2 and aflatoxin B1 were assayed using *S. typhimurium* TA98, and benzo(a)pyrene and DMBA were assayed using *S. typhimurium* TA100 in the presence of S9

Arguta-juice and deliciosa-juice significantly inhibited MeIQx mutagenicity (Fig. 2). The number of His^+^ revertants per plate found in the absence of juice was 1010 ± 63 for 60 pmol of MeIQx and the number of His^+^ revertants spontaneously formed was 42 ± 0.7 with *S. typhimurium* TA98 in the presence of metabolic activation. The amount of arguta-juice needed for 50 % inhibition (ID_50_) of the mutagenicity of MeIQx was approximately 10 μl/plate (Fig. 2a), while that of deliciosa-juice was approximately 90 μL/plate (Fig. 2b). Since a smaller amount of arguta-juice was needed to inhibit the mutagenicity of MeIQx compared to deliciosa-juice, arguta-juice was selected for further investigations of antimutagenicity. The reproducibility of the antimutagenicity toward MeIQx was confirmed using the fruit of *A. arguta* harvested in 2008 and 2010 (Fig. 2a). Arguta-juice inhibited the mutagenicity of Trp-P-2, PhIP, aflatoxin B1, benzo(a)pyrene and DMBA (Fig. 3). The number of His^+^ revertants per plate found in the absence of juice was 4592 ± 505 for 100 pmol of Trp-P-2, 877 ± 31 and for 1.0 nmol of PhIP, 1036 ± 95 for 3.0 nmol of aflatoxin B1 with *S. typhimurium* TA98 in the presence of metabolic activation, 1166 ± 189 for 50 nmol of DMBA, and 1056 ± 102 for 10 nmol of benzo(a)pyrene with *S. typhimurium* TA100 in the presence of metabolic activation. The number of His^+^ revertants spontaneously formed was 118 ± 23.5 with *S. typhimurium* TA100 in the presence of metabolic activation. The amount of arguta-juice required to achieve an ID_50_ value with Trp-P-2, PhIP, aflatoxin B1, benzo(a)pyrene and DMBA was approximately 8.0, 8.0, 15, 35 and 20 μL/plate, respectively, similar with that obtained with MeIQx.

**Fig. 4:**
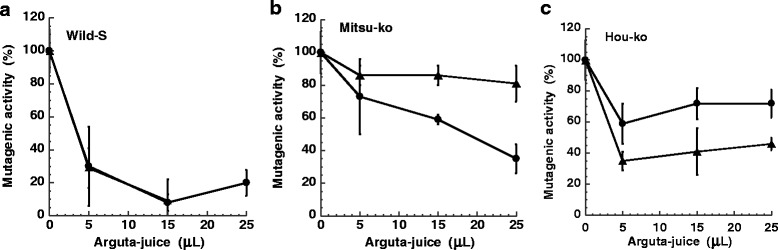
Comparison of the antimutagenicity of arguta juice between cultivars *A. arguta* ' Wild-S ' (**a**), *A. arguta* 'Mitsu-ko' (**b**), and *A. arguta* 'Hou-ko' (**c**) on the mutagenicity of MeIQx (60 pmole). Comparison between fresh samples (*triangles*) and third-day samples (*circles*) are also shown. Harvesting of the fruit of *A. arguta* was performed in 2011

The difference between cultivars of *A. arguta* (Wild-S, Mitsu-ko and Hou-ko) and the effect of preservation on antimutagenicity were examined (Fig. 4). The ID_50_ value was 3 μL/plate for the arguta-juice of Wild-S (first and third-day samples), 18 μL/plate for Mitsu-ko (third-day sample), 4 μl/plate for Hou-ko (fresh sample), and greater than 25 μL/plate for Mitsu-ko (fresh sample) and Hou-ko (third-day sample). The amount of arguta-juice of Wild-S needed for ID_50_ was lower than that of Mitsu-ko and Hou-ko both for the fresh and third-day samples. The total phenolic content was 11.1 mg/mL and 17.3 mg/mL for Hou-ko (fresh and third-day sample), 17.0 mg/mL and 15.5 mg/mL for Mitsu-ko (fresh and third-day sample), and 12.5 mg/mL and 18.7 mg/mL for Wild-S (first and third-day samples), respectively. The effect of preservation at room temperature on antimutagenicity varied in these cases.

### Effect of arguta-juice on the formation of DNA adducts in vivo

The in vivo effect of arguta-juice on the formation of DNA adducts in mice fed MeIQx was investigated. The diet-paste given to mice was completely eaten before 9 a.m. the following day. MeIQx-induced DNA adducts were formed in liver of mice fed MeIQx in their diet (Table 1 Groups 1–3). No adducts were observed in the organs of mice fed a diet mixed with arguta-juice (x3) for five days (Table 1 Group 4). The formation of DNA adducts formed in liver of mice given MeIQx in the diet decreased significantly following administration of arguta-juice (x3) compared with mice given MeIQx without juice (Table 1). No significant differences in body and liver weight or notable clinical signs of illness were observed amongst Groups 1–4 throughout the study (data not shown).

**Table 1:** In vivo effect of arguta-juice in the diet on DNA adduct formation in liver of mice fed with 0.005 % MeIQx

Group	No. of mice	MeIQx	Arguta-juice added to diet	Liver weight (g)	Adducts/ 10^8^ nucleotide
1	8	0.005 %	Water	0.81 ± 0.039	44.9 ± 39.5
2	8	0.005 %	arguta-juice (x1)	0.86 ± 0.075	35.9 ± 39.0
3	8	0.005 %	arguta-juice (x3)	0.87 ± 0.064	16.5 ± 19.5*
4	2	0	arguta-juice (x3)	0.94	0

Significantly different from Group 1 at ^*^ *p* < 0.05

### Effect of arguta-juice on the catalytic activity of CYPs, UGT and GST

**Fig. 5:**
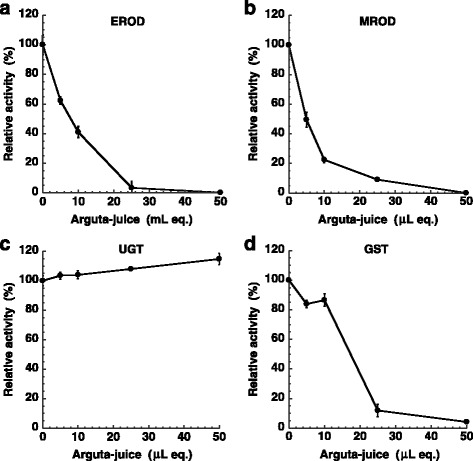
Effect of arguta-juice on CYP1A1-mediated EROD activity (**a**), CYP-1A2-mediated MROD activity (**b**), UGT activity (**c**), and GST activity (**d**). Relative activity is shown, where 100 % is defined as the activity of each enzyme in the absence of juice

In an effort to reveal the mechanisms for antimutagenicity of arguta-juice, we examined the effects on the enzymes that metabolize xenobiotics, we quantified the amount of specific metabolite produced by individual CYPs using typical substrates in the absence or presence of the juice. Arguta-juice significantly inhibited the activity of EROD (CYP1A1) and MROD (CYP1A2) (Fig. 5a and b). Relative enzymatic activity is shown in Fig. 5a, b, where 100 % is defined as the activity of CYP1A1 and CYP1A2 in the absence of juice. The ID_50_ value of arguta-juice observed when using CYP1A1 and CYP1A2 was 8.0 and 5.0 μL/mL of reaction mixture, respectively.

When arguta-juice was added to the reaction mixture of phase II enzymes, the activity of UGT increased in a dose-dependent manner to 115 % of the control (Fig. 5c), whereas the activity of GST was inhibited (Fig. 5d) and the ID_50_ value of arguta-juice with GST was 18 μL/mL of reaction mixture.

### Effect of topical application of arguta-solution on TPA-induced acute edema on the surface of mouse ears

The anti-inflammatory activity of arguta-solution was evaluated in a mouse ear model. A single topical application of TPA induced ear edema in mice. Topical application of arguta-solution inhibited TPA-induced edema of mouse ear in a dose-dependent manner (Table 2). When 20 μL of arguta-solution (x3) was applied to mouse ears prior to TPA treatment, 48.2 % inhibition of inflammation was achieved.

**Table 2:** Inhibitory effect of arguta-solution on TPA-induced inflammation of mouse ears by surface treatment. Mouse ear weight is expressed as mean ± standard deviation

Group	Topical treatment 1	Topical treatment 2	No. of ear	Average weight of ear punch (mg)	Inhibition %
1	66 % Acetone	TPA	12	18.00 ± 1.08	0
2	Arguta-solution (x1)	TPA	12	14.55 ± 0.86*	29.5
3	Arguta-solution (x3)	TPA	12	12.37 ± 1.16*	48.2
4	66 % Acetone	Acetone	4	6.31 ± 0.26	
5	Arguta-solution (x3)	Acetone	4	6.70 ± 0.44	

*P < 0.01, significantly different from the TPA-treated group (Group 1) by the *t*-test

### Partial purification and fractionation of antimutagenic and radical scavenging components in arguta-juice

Partial purification and fractionation of antimutagenic and radical scavenging components in arguta-juice was performed as outlined in Fig. 1. Based on spectrophotometric determinations, the total phenolic content in arguta-juice was 9.31 mg/ml, determined as gallic acid equivalents (Table 3). Phenolics were distributed throughout every fraction. The concentration of vitamin C in arguta-juice was 1.48 mg/mL, whereas that in 50 %-MeOH-fr was less than the detection limit (0.075 mg/mL).

**Table 3:** Partial purification of 1 l or arguta-juice; Dry weight, phenolics and biological activity in each fraction

Fraction	Dry weight	Phenolics	Antimutagenicity ID_50_ (Specific activity^b^)	DPPH scavenging activity ED_50_ (Specific activity)
	mg/mL eq.^a^ (yield)	mg/mL eq. (yield)	μL eq. (fold^b^)	μL eq. (fold)
Arguta-juice	115.3 (1)	9.31 (1)	10 (1)	10 (1)
Hexane	0.24 (0.0021)	0.181 (0.019)	(−)	(−)
-EtOAc-fr				
EtOAc-fr	3.70 (0.032)	1.20 (0.13)	(−)	(−)
100%MeOH-fr	102.3 (0.887)	2.24 (0.24)	270 (0.042)	15 (0.75)
50%MeOH-fr.	6.82 (0.059)	1.16 (0.13)	60 (2.88)	50 (3.39)
Residue-fr.	2.26 (0.020)	1.06 (0.11)	(−)	(−)

^a^ *mL eq.* mL equivalent of starting material, i.e., arguta-juice

^b^Specific activity = (ID_50_ of the original arguta-juice / ID_50_ of each fraction)/ (yield of dry weight)

**Fig. 6:**
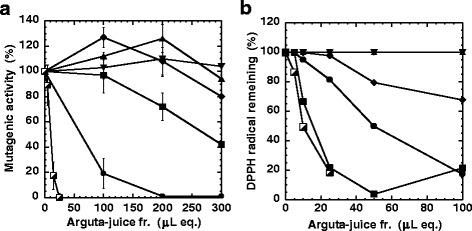
Inhibitory effect toward the mutagenicity of MeIQx (**a**) and radical scavenging activity (**b**) of each fraction. Original arguta-juice (half-closed squares), Hexane:EtOAc-fr (*upward triangles*), EtOAc-fr (*diamonds*), 100%MeOH-fr (*squares*), 50%MeOH-fr (circles), and Residue-fr (*downward triangles*)

The antimutagenic and radical scavenging activity of each fraction were examined (Fig. 6). The antimutagenic components were located in 50%MeOH-fr and 100%MeOH-fr, and the ID_50_ values when examining antimutagenicity were 60 and 270 μL eq. of arguta-juice, respectively (Table 3 and Fig. 6a). The ID_50_ value of the starting material (arguta-juice) when examining the mutagenicity of MeIQx was 10 μL, and the antimutagenicity of 50%MeOH-fr and 100%MeOH-fr was 1/6 and 1/27 that of arguta-juice, respectively. The dry weight of 100%MeOH-fr and 50%MeOH-fr was 88.7 and 5.9 % of the arguta-juice, respectively. Therefore, the specific activity determined for 50%MeOH-fr and 100%MeOH-fr with respect to antimutagenicity was 2.88 and 0.042, respectively, calculated by normalizing the activity of arguta-juice to 1 (Table 3).

The amount of DPPH radical decreased significantly in the presence of arguta-juice, as shown in Fig. 6b. The amount of arguta-juice needed to achieve a 50 % decrease in DPPH, i.e. the 50 % scavenging dose (ED_50_) was approximately 10 μl/plate (Table 3, Fig. 6b). The radical scavenging activity was also located predominantly in 50%MeOH-fr and 100%MeOH-fr (Fig. 6b). Since the ED_50_ of 50%MeOH-fr and 100%MeOH-fr was 50 and 15 μL eq. of arguta-juice, the radical scavenging activity of 50%MeOH-fr and 100%MeOH-fr was 1/5 and 1/1.5 that of arguta-juice, respectively (Table 3). The specific activity of 50%MeOH-fr and 100%MeOH-fr in terms of radical scavenging activity was calculated as 3.39 and 0.75, respectively, calculated by normalizing the activity of arguta-juice to 1 (Table 3). Hence, the specific activity in terms of both antimutagenicity and radical scavenging activity was highest in 50%MeOH-fr (Table 3).

### Antitumorigenesis study

**Fig. 7:**
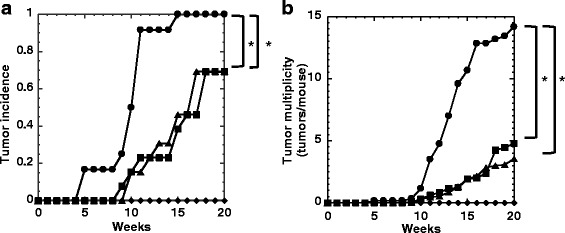
Effect of topical application of 50%MeOH-fr of arguta-juice on skin tumorigenesis in mice. **a** Tumor incidence and (**b**) tumor multiplicity were measured in female SENCAR mice treated with DMBA and TPA (Group 1, circles); DMBA, TPA and 50%MeOH-fr (x 5) (Group 2, squares); DMBA, TPA and 50%MeOH-fr (x 10) (Group 3, triangles); or DMBA and 50%MeOH-fr (x 10) without TPA (Group 4, diamonds). Each data point represents the average number per group of mice. *P < 0.01, significantly different from Group 1

Significant differences were observed concerning tumor incidence between Groups 1 and 2, and Groups 1 and 3 (*P* < 0.01) (Fig. 7a). Skin tumors appeared in the 5th week with TPA application in Group 1, whereas similar tumors appeared in the 8th and 9th weeks in groups treated with 50%MeOH-fr (Groups 2 and 3), respectively. The final mean number of tumors per mouse was 14.2, 4.8 and 3.5 for Groups 1, 2 and 3, respectively (Fig. 7b). A significant difference was observed concerning tumor multiplicity between Groups 1 and 2, and Groups 1 and 3 (*P* < 0.01). None of the mice in Group 4 developed skin tumors. No significant differences were observed with respect to animal growth and noticeable clinical signs of illness in Groups 1–4 throughout the two-stage mouse skin tumor study (data not shown).

### Effect of heat and PVPP treatment

**Fig. 8:**
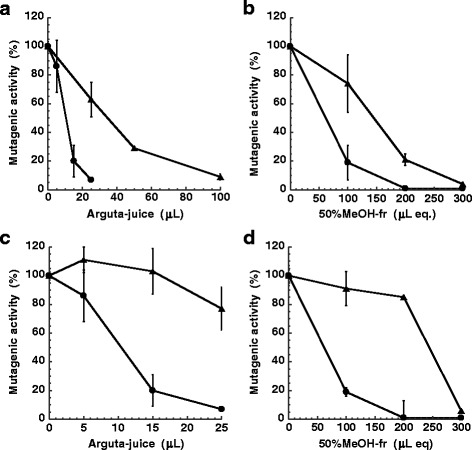
Effect of heat and PVPP treatment on the antimutagenic activity of arguta juice toward the mutagenicity of MeIQx. Effect of arguta-juice (*circle*) and heat-treated juice (*triangle*) (**a**); effect of 50%MeOH-fr (*circles*) and heat-treated 50%MeOH-fr (*triangles*) (**b**); effect of arguta-juice (*circles*) and PVPP-treated juice (*triangles*) (**c**); and effect of 50%MeOH-fr (*circles*) and PVPP-treated 50%MeOH-fr (*triangles*) (**d**)

Stability to heat was examined in an effort to investigate the nature of antimutagenic substances in arguta-juice. The antimutagenicity of arguta-juice and 50%MeOH-fr decreased following heat treatment at 90 °C for 10 min (Fig. 8a, b). Greater amounts of heated-treated arguta-juice and 50%MeOH-fr were needed to inhibit the mutagenicity of MeIQx compared to that of the original sample, which indicated that the antimutagenic substances in arguta-juice and 50%MeOH-fr comprised heat-labile compounds.

PVPP has been reported to absorb and remove phenolic compounds from solvent [31]. In an effort to determine whether the antimutagenic substances in arguta-juice comprised phenolics, arguta-juice and 50%MeOH-fr were treated with PVPP. Based on the spectrophotometric determination, the amounts of total phenolic content in arguta-juice and 50%MeOH-fr treated with PVPP were decreased to 51.2 and 38.4 %, respectively. Following PVPP treatment, ID_50_ values with respect to the antimutagenicity of arguta-juice and 50%MeOH-fr increased, and greater amounts of arguta-juice and 50%MeOH-fr were needed to inhibit the mutagenicity of MeIQx (Fig. 8c, d).

## Discussion

We investigated the antimutagenic effect of arguta-juice on well known carcinogens using the Ames test and results showed that arguta-juice inhibited the mutagenicity of heterocyclic amines (MeIQx, Trp-P-2 and PhIP), aflatoxin B1, and polycyclic aromatic hydrocarbons (benzo(a)pyrene and DMBA) (Figs. 2 and 3). Although the total phenolic content and antioxidant activity of the fruit of *A. arguta* was reported to be comparable to that of *A. deliciosa* [16], the antimutagenicity of the juice of the fruit was in the order *A. arguta* > *A. deliciosa* (Fig. 2). There was no correlation between the antimutagenicity and the amount of total phenolics of the cultivars (Fig. 4). The antimutagenic activity of the fruit of *A. arguta* differed between cultivars, and may reflect differing contents of antimutagenic components. The antimutagenicity of arguta-juice was in the order Wild-S > Hou-Ko > Mitsu-ko. Storage of the fruit at room temperature resulted in no consensus changes to the antimutagenicity. Three days after the harvest, the antimutagenicity of the arguta-juice of Wild-S showed no significant changes, whereas the antimutagenicity of the arguta-juice of Mitsu-ko increased and that of the arguta-juice of Hou-ko decreased during storage. There may be various metabolic changes that occur during storage that leads to a change in the content of antimutagenic substances.

The in vivo effect of arguta-juice was then investigated. Results showed that arguta-juice had a protective effect against DNA adduct formation in liver induced by MeIQx [32]. Addition of arguta-juice to the diet decreased the number of DNA adducts formed in liver (Table 1). Heterocyclic amines including MeIQx usually exist in cooked food, and their importance as a dietary component has been generally accepted with respect to cancer development.

Antimutagenic mechanisms were also investigated. Metabolic pathways including bioactivation and bio-inactivation are possible targets for antimutagenicity, and the inhibition of phase I enzyme activity by exogenous compounds has been observed [33]. Mutagens examined and shown in Figs. 2, 3 and 4 require bio-conversion to express mutagenicity. The principal pathways of bio-inactivation involve CYP-mediated *N*-oxidation of the exocyclic amine, CYP-mediated ring-oxidation, followed by *N*-glucuronidation with UGT [34]. Bio-activation of heterocyclic amines is known to involve *N*-hydroxylation with phase I enzymes (CYPs) followed by *O*-sulfonation or *O*-acetylation with phase II enzymes of sulfotransferase and acetyltransferase, those of aflatoxin B1 and polycyclic aromatic hydrocarbons involves ring-epoxylation with phase I enzymes. Arguta-juice decreased the activity of phase I enzymes (EROD and MROD) and GST, but not UGT. Combined effects comprising i) inhibition of the metabolic activation of mutagens with phase I enzymes, but ii) not prevented activity of phase II detoxication enzyme, UGT, for the elimination of mutagens via glucuronidation, may be responsible for the observed antimutagenicity of arguta-juice.

We also investigated the in vivo effect of the anti-inflammatory activity of arguta-solution. Tumor promotion is an essential stage of carcinogenesis and inflammation is a key factor in cancer promotion. Topical application of arguta-solution to mouse ear resulted in marked suppression of acute edema induced by TPA (Table 2). TPA causes instant irritation of mouse ear, and leads to fluid accumulation and edema, characteristic of the acute inflammatory response. Our results suggested that certain components in arguta-juice can be transcutaneously passed through to target cells and eventually lead to suppression of the inflammatory response in mouse ear.

We performed a partial purification of arguta-juice in an effort to characterize the active components. Antimutagenic and radical scavenging substances were located in the aqueous fraction and were not extracted with hexane-EtOAc or EtOAc, indicating that these substances are water-soluble (Table 3, Fig. 6). Although 88.7 % of the components in arguta-juice, calculated on the basis of dry-weight, was distributed in 100%MeOH-fr, the ID_50_ value of 50%MeOH-fr with respect to the mutagenicity of MeIQx was lower than that of 100%MeOH-fr, indicating that most of the antimutagenic substances were present in 50%MeOH-fr. The specific activity of the radical scavenging effect was also highest in 50%MeOH-fr.

We then investigated the antitumorigenic effect of topical applications of 50%MeOH-fr in the multistage carcinogenesis model in mouse skin. Topical application of 50%MeOH-fr prior to TPA treatment afforded significant protection against tumor promotion in the mouse skin tumorigenesis model (Fig. 7). Our findings showed that component(s) in 50%MeOH-fr revealed antimutagenic, radical scavenging and antitumorigenic activities.

Although one radical scavenging and water-soluble compound known in the fruit of *A. arguta* is vitamin C [12], the concentration of vitamin C in 50%MeOH-fr was less than the detection limit. Therefore, vitamin C alone may not be responsible for the observed radical scavenging and antimutagenic activity in 50%MeOH-fr. Other potential candidates present as part of the active components of arguta-juice are polyphenols. Fruit is an important source of polyphenols in the human diet [35]. The content of polyphenols in *A. arguta*, being 9.31 mg/mL, is higher than that in kiwifruit and apple (1.12 mg/g and 0.20 mg/mL of juice, respectively [15]). In arguta-juice, 12.5 % of the phenolics were present in 50%MeOH-fr (Table 3). PVPP-treatment indicated that the antimutagenic substances in arguta-juice and 50%MeOH-fr comprised phenolics (Fig. 8b, d). These results suggested that the antimutagenic components in arguta-juice and 50%MeOH-fr were water-soluble, heat-labile phenolic compounds. Further purification and identification of active ingredients will be performed in future.

## Conclusions

The present studies have demonstrated that arguta-juice inhibited the mutagenic activity of carcinogens including MeIQx, a food-borne carcinogen, and DMBA, a model compound of tumor initiation. Arguta-juice also inhibited DNA adduct formation in liver of mouse fed MeIQx, and inhibited the induction of acute inflammation of mouse ear induced by TPA, a typical promoter. Moreover, components in 50%MeOH-fr of arguta-juice led to a reduction in existing mouse skin tumors induced by DMBA-TPA. These results suggested that components in *A. arguta* are attractive candidates for potential use as chemopreventive agents.

## Abbreviations

arguta-juice, the juice obtained from the fruit of *A. arguta* squeezed with a press-squeeze; CYP 1A1, cytochrome P450 1A1; CYP1A2, cytochrome P450 1A2; DMBA, 7,12-dimethylbenz(a)anthracene; DPPH, 2,2-diphenyl-1-picrylhydrazyl; ED_50_, the amount of arguta-juice needed to achieve a 50 % decrease in DPPH; EROD, ethoxyresorufin*-O*-deethylase; EtOAc, ethyl acetate; GST, glutathione *S*-transferase; ID_50_, the amount of arguta-juice needed for 50 % inhibition; MeIQx, 2-amino-3,8-dimethyl-3*H*-imidazo[4,5-*f*]quinoxaline; MeOH, methanol; MROD, methoxyresorufin-*O*-demethylase; OD, optical density; PhIP, 2-amino-1-methyl-6-phenylimidazo[4,5-*b*]pyridine; PVPP, polyvinylpolypyrrolidone; S9, supernatant fraction of rat liver homogenate; TPA, 12-*O*-tetradecanoylphorbol-13-acetate; Trp-P-2, 3-amino-1-methyl-5*H*-pyrido[4,3-*b*]indole; UGT, 5'-diphosphoglucuronosyltransferase (UDP-glucuronosyltransferase)
